# Microarray and metabolome analysis of hepatic response to fasting and subsequent refeeding in zebrafish (*Danio rerio*)

**DOI:** 10.1186/s12864-019-6309-6

**Published:** 2019-12-02

**Authors:** Jirong Jia, Jingkai Qin, Xi Yuan, Zongzhen Liao, Jinfeng Huang, Bin Wang, Caiyun Sun, Wensheng Li

**Affiliations:** 1 State Key Laboratory of Biocontrol, Guangdong Province Key Laboratory for Aquatic Economic Animals, Guangdong Provincial Engineering Technology Research Center for Healthy Breeding of Important Economic Fish, School of Life Sciences, Sun Yat-Sen University, No.135 Xingang West Road, Guangzhou, 510275 China; 2 Present address: Key Laboratory of Sustainable Development of Marine Fisheries, Ministry of Agriculture, Yellow Sea Fisheries Research Institute, Chinese Academy of Fishery Sciences, Qingdao, 266071 China

## Abstract

**Background:**

Compensatory growth refers to the phenomenon in which organisms grow faster after the improvement of an adverse environment and is thought to be an adaptive evolution to cope with the alleviation of the hostile environment. Many fish have the capacity for compensatory growth, but the underlying cellular mechanisms remain unclear. In the present study, microarray and nontargeted metabolomics were performed to characterize the transcriptome and metabolome of zebrafish liver during compensatory growth.

**Results:**

Zebrafish could regain the weight they lost during 3 weeks of fasting and reach a final weight similar to that of fish fed ad libitum when refed for 15 days. When refeeding for 3 days, the liver displayed hyperplasia accompanied with decreased triglyceride contents and increased glycogen contents. The microarray results showed that when food was resupplied for 3 days, the liver TCA cycle (Tricarboxylic acid cycle) and oxidative phosphorylation processes were upregulated, while DNA replication and repair, as well as proteasome assembly were also activated. Integration of transcriptome and metabolome data highlighted transcriptionally driven alterations in metabolism during compensatory growth, such as altered glycolysis and lipid metabolism activities. The metabolome data also implied the participation of amino acid metabolism during compensatory growth in zebrafish liver.

**Conclusion:**

Our study provides a global resource for metabolic adaptations and their transcriptional regulation during refeeding in zebrafish liver. This study represents a first step towards understanding of the impact of metabolism on compensatory growth and will potentially aid in understanding the molecular mechanism associated with compensatory growth.

## Background

Long-term fasting may cause growth retardation and severe damage in fish. To overcome the negative effects of food shortage, metabolic flux is modified [1–3]. When the food supply is restored, some species can accelerate their growth and promote biomass accumulation, which is called compensatory growth [4]. The nervous system, liver and muscle participate in compensatory growth in different ways. For example, most fish undergoing compensatory growth develop an enormous appetite, which is regulated by neuropeptides such as orexin, neural peptide Y (NPY) and agouti gene-related protein (AgrP) in the central nervous system [5, 6]. Restoring food intake after fasting increases the expression of growth hormone receptor in the liver, improving the sensitivity of liver tissue to growth hormone. Liver IGF1 (insulin-like growth factor 1) secretion is then activated, which plays important roles in growth and anabolic metabolism [7, 8]. During refeeding, the expression of the muscle-specific ubiquitin ligases MAFbx and MuRF1 are downregulated, thereby reducing muscle tissue protein degradation [9, 10]. As muscle tissue growth is determined by the balance of protein synthesis and protein breakdown, a reduction in protein degradation may be one of the reasons for the increase in total muscle mass during compensatory growth. In our earlier studies, liver-derived reactive oxygen species have been shown to regulate muscle fiber growth in a way that has not been elucidated [11]. Therefore, we speculated that liver metabolism was involved in compensatory growth, which was why liver was chose for the following analysis.

Considering the complexity of compensatory growth, omics approaches are good tools to study the molecular mechanism of compensatory growth. According to the report by Connor et al. [12], liver microarray analysis of cattle that resumed feeding for 1 day after 2 weeks fasting showed that oxidative phosphorylation, the tricarboxylic acid cycle, purine and pyrimidine metabolism, carbohydrates, fatty acid and amino acid metabolism, as well as glucose metabolism were upregulated. The author hypothesized that compensatory growth was caused by a combination of lower basal metabolism and enhanced mitochondrial function. Rescan et al. investigated the gene expression changes in salmon muscle tissues recovered at day 4, 7, 11 and 36 days after fasting for 30 days [13]. The microarray results showed that mRNA synthesis, translation, protein folding and maturation, ribosome formation, oxidative phosphorylation and DNA replication pathways were upregulated after recovery for 11 days. Another study focused on the recovery of trout muscle tissues 4, 11 and 36 days after refeeding; the results showed that the compensatory growth process upregulated transcription, RNA metabolism and mitochondrial functions [14].

Teleost fishes represent a highly diverse group consisting of more than 20,000 species. Many fish species have the ability to gain the weight of continuously fed fish after a period of restricted feeding [15, 16]. Identifying the mechanism of compensatory growth would assist in the selection of animals with improved feed efficiency, thereby reducing the overall costs of animal farming. Here, we confirmed zebrafish to be a suitable model for a compensatory growth study. After 3 weeks of fasting, feeding recovery for 3, 10 and 15 days were chosen as initial, middle and late sampling sites, respectively, during a compensatory growth study, and liver samples from each time point were applied for microarray analysis. At the same time, liver samples from zebrafish fed ad libitum as well as those fasted for 3 weeks were also selected for microarray analysis. The liver metabolome was examined after refeeding for 3 and 10 days after 3 weeks of fasting to better understand the metabolic adjustment during compensatory growth in zebrafish.

## Results

### Influence of fasting and refeeding on the body weight and histomorphology of liver tissues

**Fig. 1:**
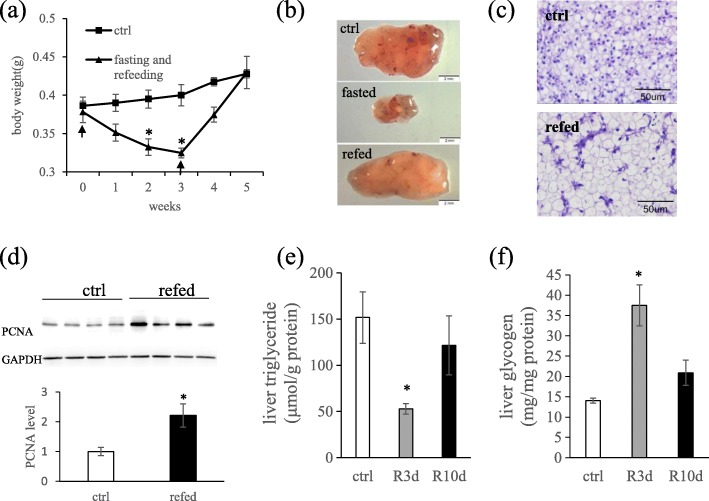
Effects of fasting and refeeding on zebrafish body weight and hepatocyte morphology. **a** Growth curve of zebrafish during fasting and refeeding. Arrows represent the start of fasting and refeeding, respectively; asterisks denote significant differences between fasting and the control group at the same stage (*P* < 0.05), *n* = 5–6. **b** Representative gross liver tissues from zebrafish fed ad libitum (ctrl), fasted for 3 weeks (fasted) and re-fed 3 days after a 3-week fast (refed). Scale bar, 2 mm. **c** H&E staining of liver samples from fed ad libitum (ctrl) and re-fed for 3 days following a 3-week starvation (refed). **d** Western blot analysis of PCNA expression in liver of zebrafish fed ad libitum (ctrl) and re-fed 3 days after a 3-week fast (refed), *n* = 4. **e** Triglyceride (TG) content in zebrafish liver when refed for 3 days (R3d) and 10 days (R10d) after a 3-weeks fasting, *n* = 4. **f** Glycogen content in zebrafish liver when refed for 3 days (R3d) and 10 days (R10d) after a 3-weeks fasting, *n* = 4–6. Error bars were ± SEM. For d, e and f, asterisks denote significant differences between refed group and the control group (*P* < 0.05)

No significant difference in initial body weight was found between the control and fasted groups (*P* > 0.05). Zebrafish fasted for 3 weeks lost 26% of their body mass, and significant differences in body weight were observed from 2 weeks of starvation (*P* < 0.05). Upon refeeding, the food-restricted zebrafish showed a higher weight gain ratio than the continuously fed fish (32.2% versus 7.0% in the refed 2 weeks), and caught up to the final body weight in approximately 2 weeks (Fig. 1a). Thus, we selected refeeding for 3, 10 and 15 days to represent early, middle and late phase compensatory growth, respectively. The liver size of zebrafish is greatly influenced by nutrition status, as fasting reduced liver size, while refeeding resulted in hepatomegaly (Fig. 1b). Refeeding for 3 days after 3 weeks of fasting caused a moderate increase in hepatocyte size (Fig. 1c), while the protein levels of proliferating-cell nuclear antigen (PCNA), a marker of cell proliferation [17], was significantly increased compared with the fish not undergoing fasting (Fig. 1d). The liver is considered to be the main lipogenic tissue in fish [18]. The lipid contents in livers of refed zebrafish were observed using a TG reagent kit. The results showed decreased TG contents after refeeding for 3 days and similar TG contents to the control group after refeeding for 10 days (Fig. 1e). The glycogen contents in the liver were increased after refeeding for 3 days and gradually restored after refeeding for 10 days (Fig. 1f).

### Temporal transcriptome during zebrafish compensatory growth: overview

**Fig. 2:**
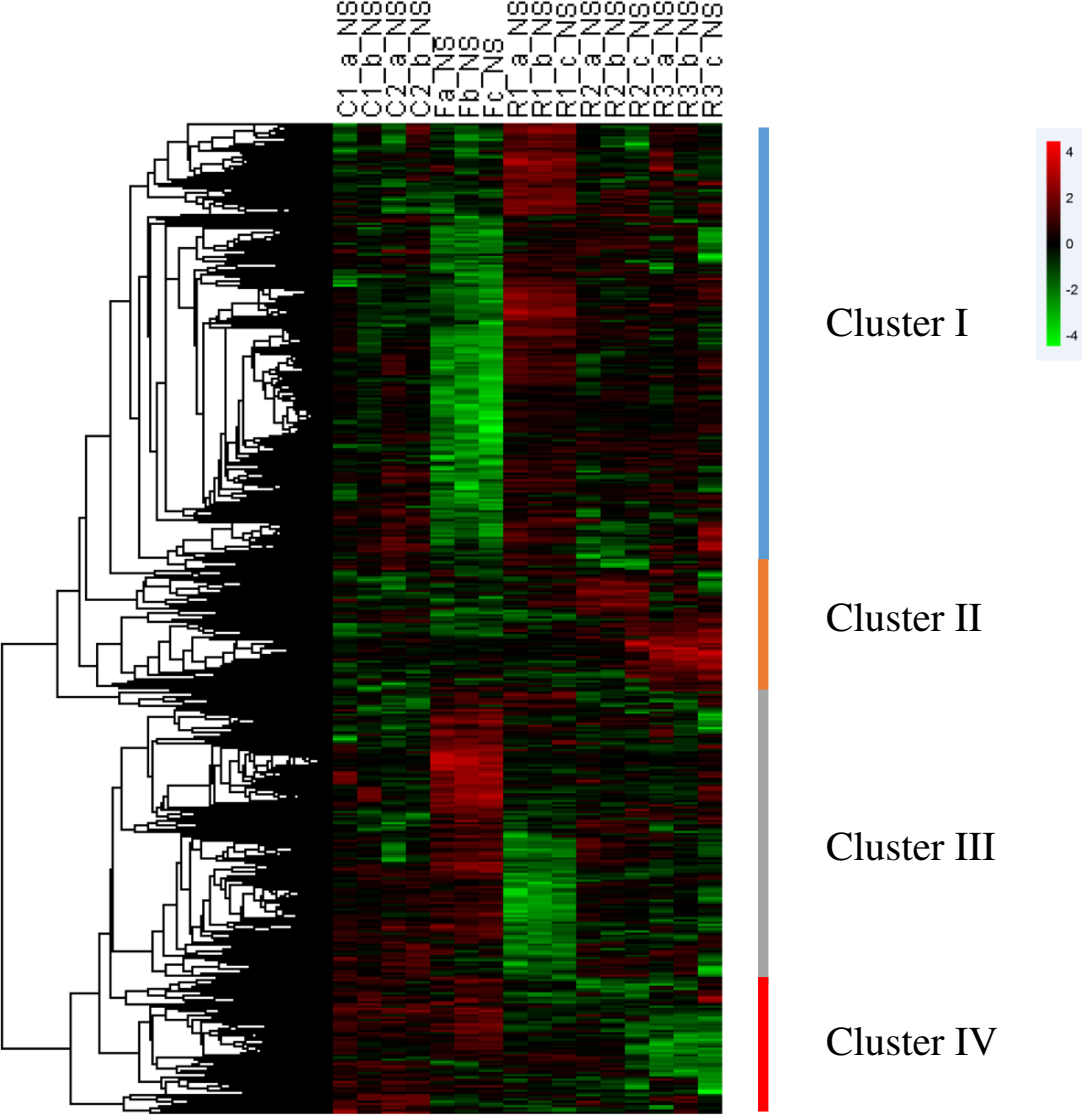
Hierarchical clustering of differentially expressed genes during fasting and refeeding in zebrafish liver. Unsupervised clustering of differentially expressed genes led to the formation of four distinct clusters (I, II, III and IV). Each row represented the temporal expression pattern of a single gene and each column represented a single sample. Columns 1 to 4, liver samples from continuously fed group; columns 5 to 7, liver samples at fasted for 3 weeks; columns 8 to 10, liver samples at day 3 after refeeding; columns 11 to 13, liver samples at day 10 after refeeding; columns 14 to 16, liver samples at day 15 after refeeding. The expression levels were represented by colored tags, with red representing higher levels of expression and green representing lower levels of expression

ANOVA testing (Benjamini-Hochberg corrected *p*-values < 0.05) and a fold change threshold of 2 while q-value threshold of 0.1 were used to define genes with expression levels that were significantly different at the different stages of sampling compared to zebrafish fed ad libitum. This led to the identification of approximately 4000 unique differentially expressed genes that were then hierarchically clustered. The unsupervised clustering, which is shown in Fig. 2 and is available using the heat map file and Java treeview tool (https://sourceforge.net/projects/jtreeview/files/), resulted in the formation of four major gene clusters that displayed the following distinct temporal profiles: clusters I and III were composed of genes with opposite expression patterns between the fasted and 3 days of refeeding groups, gradually recovered with later refeeding; cluster II was composed of genes specifically overexpressed at 10 or 15 days of refeeding; and cluster IV was composed of genes that were downregulated during refeeding, while not participated in the fasting. Several genes not belonging to any of these four clusters were abandoned in our further analysis.

### Genes upregulated after 3 days of refeeding

Cluster I contained 1852 unique genes with early and transient induction after refeeding for 3 days after 3 weeks of fasting, and most of these genes recovered their expression after refeeding for 15 days. In total, 1694 genes from cluster I were eligible for analysis using the DAVID (Database for Annotation, Visualization and Integrated Discovery) software tools and were subsequently used for functional analysis. Gene Ontology of cluster I using DAVID revealed a very high enrichment of functional categories related to DNA repair (GO:0006281, *P* < 3.37 × 10^− 8^, 38 genes) and cell cycle (GO:0007049, *P* < 4.04 × 10^− 8^, 37 genes), indicating that cell proliferation occurred early in refed zebrafish liver. Some metabolic processes were also clustered, such as lipid metabolic process (GO:0006629, *P* < 2.26 × 10^− 4^, 32 genes), fatty acid biosynthetic process (GO:0006633, *P* < 4.96 × 10^− 4^, 12 genes) and ATP metabolic process (GO:0046034, *P* < 0.0099, 5 genes). Several genes belonging to the glycolytic pathway were also upregulated in R1, even though *P* > 0.05. At the same time, cell redox homeostasis (GO:0045454, *P* < 3.31 × 10^− 4^, 15 genes) and ubiquitin-dependent protein catabolic process (GO:0006511, *P* < 0.0072, 18 genes) were also upregulated after refeeding for 3 days. For details, see Additional file 1 for lists of genes that composed the major functional categories in cluster I.

### Genes upregulated at 10 days or 15 days post-refeeding

Cluster II included approximately 467 unique genes specifically upregulated at 10 or 15 days after refeeding began. DAVID analysis of the 379 eligible genes showed that cluster II was highly enriched in genes involved in response to stimulus (GO:0050896, *P* < 0.0026, 10 genes), G-protein coupled receptor signaling pathway (GO:0007186, *P* < 0.011, 26 genes), smoothened signaling pathway (GO:0007224, *P* < 0.011, 4 genes), fibroblast growth factor receptor signaling pathway (GO:0008543, *P* < 0.022, 4 genes), etc. Several odorant receptors were upregulated during later refeeding, such as *or126–4*, *or115–13*, *or103–4*, *or116–1*, *or111–7*, *or109–1*, and *or108–2*. For details, see Additional file 2 for the lists of genes that composed the major functional categories in cluster II.

### Genes upregulated during fasting that recovered during refeeding

Cluster III contained approximately 1203 unique genes upregulated during fasting and downregulated when food was resupplied. When refeeding was sustained, the expression of these genes returned to the level of the control group. DAVID analysis performed on 1042 eligible genes indicated that this cluster was enriched in genes encoding the steroid hormone-mediated signaling pathway (GO:0043401, *P* < 1.83 × 10^− 5^, 15 genes), arachidonic acid metabolic process (GO:0019369, *P* < 2.88 × 10^− 4^, 6 genes), regulation of insulin-like growth factor receptor signaling pathway (GO:0043567, *P* < 6.46 × 10^− 4^, 5 genes) and negative regulation of cell proliferation (GO:0008285, *P* < 0.0016, 9 genes). Additionally, several nuclear receptors were also found in this cluster, such as *nr0b1*, *nr1d2a*, *nr5a5*, *nr1d1*, *nr1h4*, *nr1i2*, *retinoid X receptor*, *alpha a (rxraa)*, *retinoid X receptor*, *gamma b (rxrgb)*, and *thyroid hormone receptor beta (thrb)*. For details, see Additional file 3 for the lists of genes that composed the major functional categories in cluster III.

### Genes with a tendency for downregulation during early and late refeeding

Cluster IV included more than 368 unique genes that were downregulated early or late during the refeeding experiment. The DAVID analysis of 303 eligible genes showed that cluster IV was highly enriched in genes involved in calcium ion transport (GO:0006816, *P* < 0.017, 5 genes), gluconeogenesis (GO:0006094, *P* < 0.029, 3 genes) and thyroid hormone generation (GO:0006590, *P* < 0.042, 2 genes). For details, see Additional file 4 for the lists of genes that composed cluster IV functional categories.

### Validation of the microarray gene expression data

**Fig. 3:**
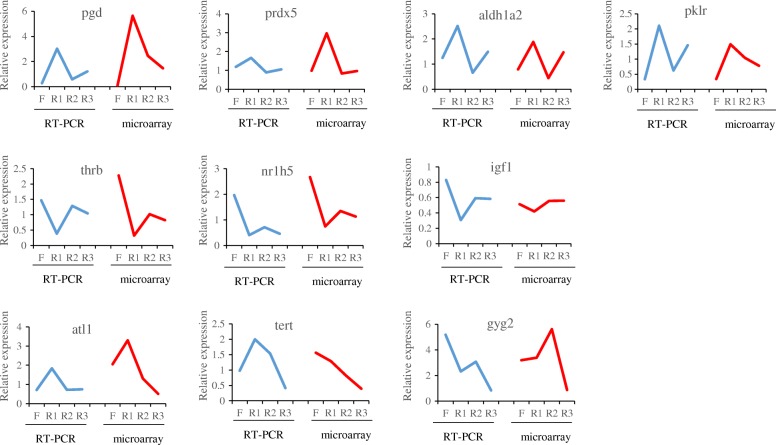
Comparison of RT-PCR and microarray expression ratios for selected genes. Blue curves represented results from RT-PCR, *n* = 8–10; red curves represented results of microarray results. F, R1, R2 and R3 represented liver samples of fasted for 3 weeks, refed for 3 days, refed for 10 days and refed for 15 days

To confirm the significance of the differential mRNA expression patterns observed in the microarray data, real-time PCR analysis was performed on selected genes that exhibited distinct temporal profiles during fasting and refeeding. Among the ten tested genes, *pgd*, *prdx5*, *aldh1a2* and *pklr* belonged to cluster I; *thrb*, *nr1h5* and *igf1* belonged to cluster III; and *atl1*, *tert* and *gyg2* belonged to cluster IV. The temporal expression patterns of these genes revealed by microarray and real-time PCR data were very similar (Fig. 3). Pearson correlations between the differences in expression measured by quantitative real-time PCR and microarray were greater than 0.70, except for *igf1* (*r* = 0.629), *tert* (*r* = 0.488) and *gyg2* (*r* = 0.486).

### Impacts of refeeding on zebrafish liver metabolomics

**Fig. 4:**
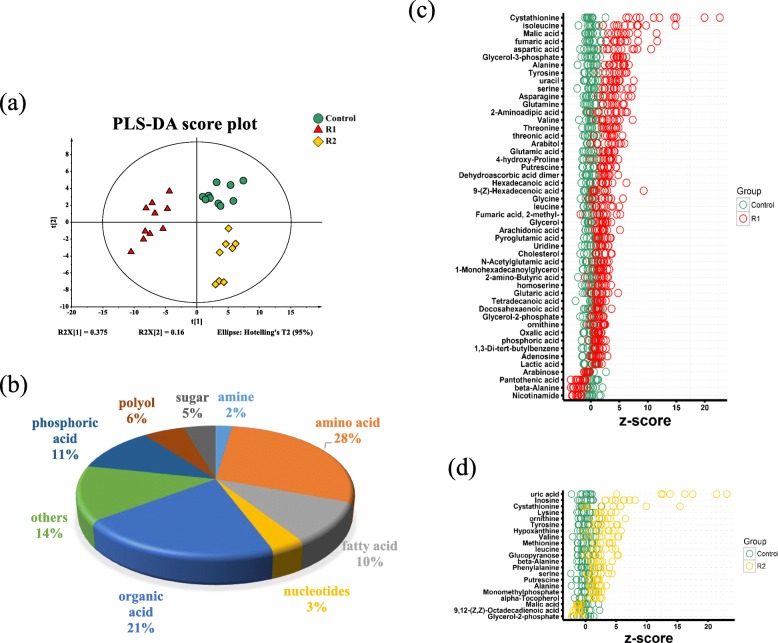
Metabolic profiles of zebrafish liver during refeeding. **a** Score plot of the PLS-DA model from all detected metabolites. **b** Category of detected metabolites. **c** Z-score scatter diagrams of differential metabolites in R1 (refed 3 days) period based on control. **d** Z-score scatter diagrams of differential metabolites in R2 (refed 10 days) period based on control. For **c** and **d**, the data from tested groups were separately scaled to the mean and standard deviation of control. Each point represented one metabolite in one technical repeat and was colored by sample types

To better validate the microarray results and understand the metabolome changes associated with refeeding, untargeted metabolomics was performed on zebrafish liver after refeeding for 3 days (R1) and 10 days (R2) after 3 weeks of fasting using the GC-MS platform. According to the original principal-component analysis (PCA) scores, two samples from the R2 group were excluded from the analysis. The PLS-DA (Partial least squares discrimination analysis) score was recalculated and is shown in Fig. 4a. The data from each group were in a 95% confidence interval, and good clustering was shown within the group. There was also a good distinction between groups, indicating differences in the metabolite contents among the different time points. Among the 88 detected metabolites, 28, 21, 11, and 10% belonged to amino acid, organic acid, phosphoric acid, and fatty acid, respectively. Polyol, sugar, nucleotides and amine were also detected with a smaller proportion (Fig. 4b).

Using statistical cut-offs such as a *P*-value < 0.05 and fold change > 1.5 or < 0.667, 45 metabolites were upregulated and 4 were downregulated among the R1 samples (Additional file 5: Table S1); the Z-score displaying the variations in these metabolites is shown in Fig. 4c. Among the differential abundance of metabolites, amino acids were the most significant. Apart from β-alanine, phenylalanine, proline, lysine, cysteine and methionine, all the other detected amino acids had elevated abundances in R1. Refeeding for 3 days showed increased levels of glycerol-3-phosphate (3PG), glycerol-2-phosphate (2PG) and lactate, all of which are glycolytic intermediates. At the same time, the levels of some fatty acids, such as 9-(Z)-hexadecenoate, arachidonic acid, docosahexaenoic acid, myristoic acid and palmitic acid, were significantly increased during the R1 period. The abundance of lactate, fumarate and malate during the R1 stage was higher than in the control group, indicating the reinforced TCA cycle during early refeeding.

Using statistical cut-offs such as a *P*-value < 0.05 and fold change > 1.5 or < 0.667, 18 metabolites were upregulated and 3 were downregulated among the R2 samples (Additional file 5: Table S2); the Z-score displaying the variations in these metabolites is shown in Fig. 4d. The most significantly accumulated metabolite in the R2 liver was uric acid. The types of metabolites with the most significant changes in concentration were still amino acids. The abundance of malate, which was increased in R1, was reduced in R2, suggesting the restoration of the TCA cycle during R2.

**Fig. 5:**
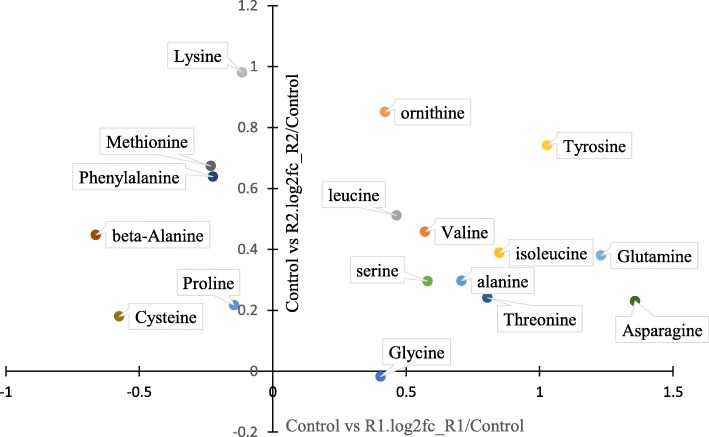
Scatter diagram of amino acid levels comparing to control group during R1 (refed 3 days) and R2 (refed 10 days) period

**Fig. 6:**
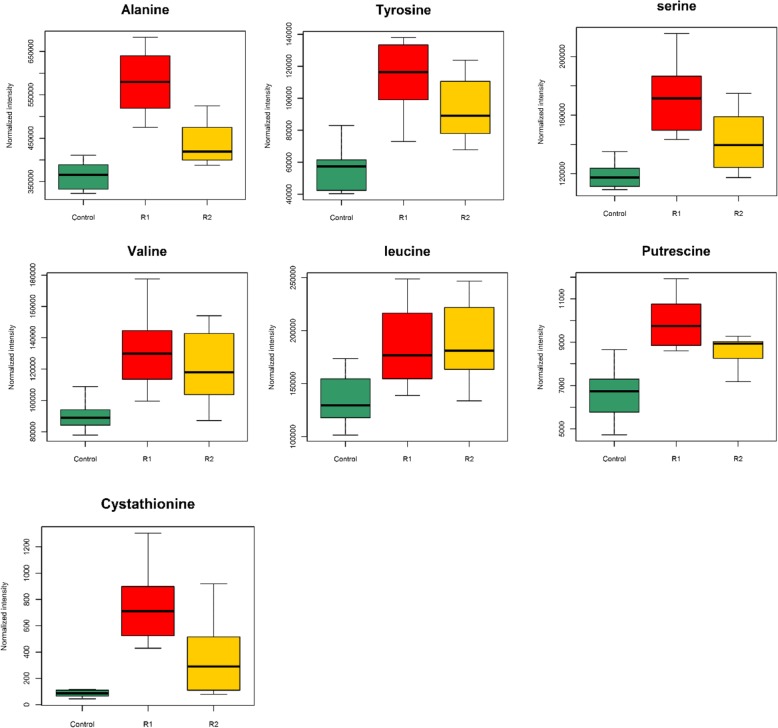
Boxplots represented metabolites differences in R1 (refed 3 days) and R2 (refed 10 days) period comparing to the control group

We summarized the amino acid concentration variations during R1 and R2 in Fig. 5, and found that except for glycine, the concentrations of other amino acids were increased to some extent during R2. Metabolites that had persistently high abundance during R1 and R2 were also emphasized (Fig. 6), except for five amino acids. Putrescine and cystathionine were found to accumulate in the liver during early and late refeeding; both of these compounds are amino acid metabolites [19, 20].

## Discussion

### Gene expression alterations during fasting

When zebrafish were fasted, the loss of body weight was the most prominent in the first week, and slowed down in the subsequent weeks. This adaptation to starvation was also found in other species [21]. Fasting is usually characterized by decreased cellular metabolism and reduced thyroid hormone (TH) concentration in plasma [7, 22], but the regulation of the TH system in peripheral tissues appears to be complicated. The triiodothyronine (T3) content was significantly increased with reduced type I deiodinase (DIO1) and increased type 3 deiodinase (DIO3) in mice livers after 28 and 36 h of fasting [23]. In prolonged fasted northern elephant seal pups, the mRNA levels of *dio1*, *dio2* and *thyroid hormone receptor b* (*thrb*) were increased in muscle and adipose [24], which was called “adaptive fasting” by the authors [22]. According to our microarray results, the mRNA expression of *thrb* and *dio2* were increased in the liver after 3 weeks of fasting, while *dio1* expression was decreased. TH has recently been shown to couple autophagy with mitochondrial fat oxidation and the induction of ketogenesis in the liver [25], while autophagy and ketogenesis are common responses to starvation [26, 27]. The expression changes in the thyroid hormone system during fasting suggested its important roles in fasting adjustment in zebrafish.

Several articles have confirmed the participation of nuclear receptors during fasting; for example, farnesoid X receptor (FXR, encoded by *nr1h4*) protects liver cells from apoptosis induced by fasting [28] and regulates triglyceride and carbohydrate metabolism at the same time [29, 30]. Our microarray results showed that several nuclear receptors were upregulated in the fasted state and gradually returned to basal levels during refeeding. The microarray results provide a valuable resource for further analysis of nuclear receptor genes that are potentially involved in fasting adaptation.

### The involvement of energy metabolism in early refeeding

The fastest growth was achieved in the first week of refeeding, accompanied by significant variation in transcriptomics and metabolomics. Therefore, the initial phase of refeeding might be key to understanding compensatory growth [31]. The intermediary metabolites in glycolysis, such as 3PG, 2PG, and lactate, an end product of glycolysis, were shown to be increased during R1, which indicated increased glycolytic flux [32]. The increase in the gene expression of the rate-limiting enzyme in glycolysis, *pyruvate kinase, liver and RBC (pklr)*, also verified the booster effects of refeeding on glycolytic flux. Gluconeogenesis-related genes were downregulated, such as *fructose-1,6-bisphosphatase 2* (*fbp2*), *glucose-6-phosphatase b*, *catalytic subunit* (*g6pcb*) and *pyruvate carboxylase b* (*pcxb*), indicating reduced hepatic gluconeogenesis in early refeeding, which was supported by other studies [33].

Glycogen accumulated in the liver during early refeeding (R1), while the expression of glycogen metabolism-related genes was not altered [such as *glycogen synthase 2* (*gys2*) and *phosphorylase, glycogen, liver* (*pygl*)], which implied regulation of glycogen metabolism in the liver was transient and may occur through a transcription-independent pathway. Glycogen accumulation caused by refeeding was reported in mice [34], though in most fish, glycogen levels were just restored to the control level during refeeding [35, 36]. In rats, the glycogen content was associated with liver cell size [37], and similar correlations were found in zebrafish. Gene Ontology (GO) analysis enriched for lipid metabolic process (GO:0006629) and fatty acid metabolism (GO:0006636, GO:0042759) in R1, in addition to fatty acid and 3PG accumulation in the liver, which indicated the participation of lipid biosynthesis in early refeeding [38]. It appears that the newly synthesized lipids in the liver were exported to other peripheral tissues for storage, as enhanced lipid metabolism did not promote an increase in the hepatic lipid contents.

According to the microarray results, oxidative phosphorylation activities, especially the F_0_-F_1_ ATP synthase complex (GO:0015986, ATP synthesis coupled proton transport) was upregulated after refeeding for 3 days; meanwhile, the accumulation of fumaric acid and malic acid in the liver indicated the elevated TCA cycle function. The improvement in the TCA cycle and oxidative phosphorylation were also observed in mammals during compensatory growth [12] or catch up fat [39]. Attempts to assess the wide gene expression regulation of OXPHOS by environmental stressors, such as hypoxia, temperature and nutrition have been addressed in several fish. For example, both starvation and chronic cold-thermal stress upregulated OXPHOS genes in gilthead sea bream [40, 41], which enables maintaining homeostasis and survival. GO analysis showed that cell redox homeostasis (GO:0045454) was enriched during R1, which is a protective mechanism to prevent oxidative damage [42, 43], and the enhanced OXPHOS activity might be necessary for the adaptation. On the other hand, compensatory growth is accompanied with anabolism and biomass accumulation, which is an energy requiring process [44]. The ubiquitin-dependent protein catabolic process, which was up-regulated in the early refeeding, also required ATP for its function [45]. The presence of these numerous ATP driven steps during compensatory growth indicates the importance of mitochondrial function improvement. Tilapia (*Oreochromis niloticus*) were fasted and refed and established as a negative model of compensatory growth in our lab, and such metabolism adjustment was missing after 2-weeks’ fasting and 3 days of refeeding (data not shown). So, we believe that there is a certain correction between the TCA-OXPHOS axis and compensatory growth, but how this happens and whether it might be important has not been studied in detail.

Amino acids accumulated in the liver during refeeding, but the changes in each amino acid were not consistent. For example, the concentrations of methionine, cysteine, lysine, phenylalanine, valine, and β-alanine were decreased during R1. Amino acid accumulation in the liver may originate from food or protein breakdown since the proteasome function was improved during early refeeding; these amino acids could serve as precursors for protein synthesis or be consumed to produce metabolites with biological activity [46]. The differences in the amino acid concentrations during R1 may be caused by different degrees of consumption. At the same time, these decreased amino acids have been shown to play important roles in fish growth and can be classified as functional amino acids [46]. For example, additional methionine can influence growth and lipid metabolism [47], while lysine can be converted into carnitine and promote growth and fatty acid oxidation [48]. The accumulation of putrescine and cystathionine, which are derived from arginine- or proline-derived ornithine [49] and methionine [50], respectively, also verified the metabolism of amino acids in the liver during compensatory growth.

Both the microarray and Real time PCR results confirmed the down-regulated expression of igf1, which was also proved by several articles [51–53]; on the other hand, several researches have proved the participation of GH-IGF systems in compensatory growth [8, 54]. We speculated that IGF-I is not critical for activation of compensatory growth in zebrafish.

### Pathways and components involved in later refeeding

There were fewer pathway and metabolite differences in R2 and R3 compared to R1. It appears that the liver could gradually recover after 2 weeks of refeeding. The expression of several odorant receptors (ORs) were increased during later refeeding. Odorant receptors belong to the superfamily of G protein-coupled receptors (GPCRs) and are primarily expressed in the olfactory epithelium [55]. ORs are also found in several nonolfactory tissues, such as adipose, liver and intestine [56]. For example, activation of OR1A1 can suppress PPAR-γ expression in cultured hepatocytes and modulates hepatic triglyceride metabolism [57]. ORs can sense small organic molecules and activate pathways involved in survival and proliferation, such as the MAPK, Rho and AKT signaling cascades [58]. Some ORs are even overexpressed in tumor cells [59]; thus, the functions of ORs in the liver during refeeding should be investigated in future studies.

We also observed an accumulation of uric acid in the liver after 10 days of refeeding. Uric acid can act as a pro- and antioxidant and activator of the immune response, as well as regulate glucose and lipid metabolism under different situations [60, 61]. Thus, the exact cause and role of uric acid accumulation in later refeeding should be explored in following research.

## Conclusion

In the present study, integrated metabolome and transcriptome analysis of zebrafish liver samples provided an overview of the global metabolic adaptations in the liver during compensatory growth. These data confirmed the existing research results to some extent and provided new research ideas for the study of compensatory growth. The activation of glycolysis and oxidative phosphorylation in the early stage of refeeding seemed to be important for the stimulation of compensatory growth. This metabolic reprogramming was suggested to be a biomarker for the compensatory growth.

## Methods

### Fish culture and experiment station

Female zebrafish that approximately 3 months old were bought from the local aquarium market, randomly allocated to tanks (24 L capacity) with stocking density of 30 individuals per tank. In order to avoid sex-specific metabolism, only female fish were selected for the experiment. The tanks were supplied with flow-through water at 28 ± 1 °C and a photoperiod maintained at constant 12 h/day (8 a.m.-8 p.m.). Following a 2-weeks acclimation period, the zebrafish were starved for 3 weeks. When the fasting ended, commercial fish food pellet was re-supplied twice daily (9:00 a.m. and 4:00 p.m.). Each treatment contained of three replicates, body weight was detected weekly during fasting and 2 weeks refeeding, and weight gain ratio (WGR, %) was calculated as the formula bellow: (average final body weight -average initial body weight)/ total initial body weight*100. In order to rule out the impact of metabolic rhythm on the results, liver tissues were collected at 3 p.m., so did in the fasting group. All fish rearing were performed at the Fish culture experiment station in Sun Yat-sen University, Guangdong Province, China.

### Hematoxylin and eosin (H&E) staining

H&E staining was performed as previously described [62]. After euthanizing the zebrafish, fresh liver from each fish was separated rapidly, fixed in Bouin’s Fluid, and dehydrated using grades of ethanol (70, 80, 90, 95, and 100%). Dehydration was followed by clearing the samples in two changes of xylene. The samples were then impregnated with two changes of paraffin, embedded, and blocked out. The tissues were cut into 5-μm sections by microtome and stained with hematoxylin and eosin (Beyotime, China). The sections were then observed and photographed with an optical microscope (Olympus, Japan).

### Determination of hepatic lipid accumulation

Hepatic TG level was measured by a colorimetric array (Dongou, China) as previously reported [63]. Liver tissues were homogenized in lysis buffer, the homogenate was centrifuged at 2000 g for 10 min and the supernatant was collected. The liver lysate was quantified with BCA Protein Assay Kit (Beyotime, China) and 20 μg protein was reacted with 200 μl reaction buffer. The reaction was sustained for 10 min at 37 °C, and absorbance was quantified at 570 nm. All samples were determined in duplicate and triglycerides values were represented as μmol of triglycerides/g of protein.

### Hepatic glycogen content detection

The content of liver glycogen was determined with a glycogen assay kit using the anthrone reaction method (Comin, China). Briefly, zebrafish livers were homogenized in 600 μl glycogen hydrolysis buffer. Samples were centrifuged at 8000 g for 10 min and the supernatant was collected. 240 μl anthrone-H2SO4 mix were added to 60 μl of samples and incubated at 95 °C for 10 min. After the incubation, 200 μl supernatant was transferred to a 96 well plate, absorbance at 620 nm was measured. The glycogen content was expressed as milligrams of glucose equivalents per milligrams of liver protein.

### Western blot

Western blot analysis was generally performed as described previously [64]. Briefly, liver samples were homogenized by a Bullet Blender Tissue Homogenizer (Next advance, USA) in RIPA Lysis Buffer (Beyotime, China). The protein concentration was quantified using the BCA assay (Beyotime, China) according to the manufacturer’s instructions. Twenty micrograms of protein was subjected to SDS-PAGE and transferred to a PVDF membrane (Merck, USA) through electroblotting. After being blocked with non-fat dry milk, the membranes were washed with TBST and then incubated overnight at 4 °C with anti-PCNA (Santa Cruz Biotechnology, USA) or anti-GAPDH (Cell Signaling Technology, USA) antibody. The next day, after three 10-min washes with TBST, the membranes were incubated for 1 h at room temperature with HRP-conjugated goat anti-rabbit IgG antibody (Boster, China). After another three 10-min washes, the membranes were visualized using an enhanced chemiluminescence (ECL) detection kit (Tanon, China). Quantification the intensity of western blot binds was carried out by using ImageJ software.

### Microarray

Liver samples were obtained at the end of fasting trial and named as the F group. During the the refeeding period, zebrafish livers were sampled at 3, 10, and 15 days as R1, R2 and R3, in order to assess long-term changes in the assessed molecules. The control group were fed throughout and liver samples were both obtained before food withdrawn and at the end of refeeding. Total liver RNA was prepared with TRIzol according to the manufacturer’s protocol (Life Technologies, USA). The RNA samples were sent to Shanghai Biotechnology Corporation for customer cDNA microarray analysis. RNA quantity and purification was determined by optical density measurements (OD260/280) and RNA integrity by using the bioanalyzer 2100 (Agilent technologies, USA). Total RNA was amplified and labeled by Low Input Quick Amp Labeling Kit, One-Color (Agilent technologies, USA). Labeled cRNA were purified by RNeasy mini kit (QIAGEN, Germany). Microarray experiments were performed using an Agilent-based microarray platform with 4 × 44 K probes per slide (GEO platform record: GPL14664). Each slide was hybridized with 1.65 μg Cy3-labeled cRNA using Gene Expression Hybridization Kit (Agilent technologies, USA) in Hybridization Oven (Agilent technologies, USA), according to the manufacturer’s instructions. After 17 h hybridization, slides were washed in staining dishes (Thermo Shandon, USA) with Gene Expression Wash Buffer Kit (Agilent technologies, USA). Slides were scanned by Agilent Microarray Scanner (Agilent technologies, USA). Data were extracted with Feature Extraction software 10.7 (Agilent technologies, USA). Raw data were normalized by Quantile algorithm, Gene Spring Software 11.0 (Agilent technologies, USA).

### Bioinformatic analysis

In order to identify the differentially expressed genes in the fasting and refeeding group compared with the normal feeding group, gene signal intensities with a fold-change of < 0.5 or > 2.0, *p*-value < 0.05 and q-value < 0.1 were selected. For the clustering analysis, the data were median-centered and an average linkage clustering was carried out using CLUSTER software. The results were visualized using TREEVIEW. GO enrichment analysis was performed using Database for Annotation, Visualization and Integrated Discovery (DAVID) [65].

### Quantitative real-time PCR

RNA extraction and complementary DNA generation were performed as described previously [66]. A total of 10 μL of the PCR reaction volume contained 5 μL of SYBR® Green Realtime PCR Master Mix (TOYOBO, Japan), 0.6 μL of forward and reverse primers (10 μM each), 1 μL of 10-fold diluted cDNA templates and 3.4 μL of water. Primers used in real-time PCR are shown in Additional file 6. Amplification of samples were carried out with the Roche LightCycler 480 Real-time PCR Detection System using the following thermal cycling profiles: 95 °C for 90 s, 40 cycles of 95 °C for 20 s, 55 °C for 15 s, and 72 °C for 15 s. Samples without RT were run for each reaction as negative controls. The 18S rRNA was used as the internal reference, and the 18S levels remained stable between various treatments throughout the study. The relative gene expression levels were normalized to the 18S levels and were calculated by the comparative Ct method. Pearson correlations were computed to compare the expression values of microarray and quantitative real-time PCR.

### Metabolite extraction and metabolite profiling analysis

Based on the chip results, the early stage of refeeding might be the most important period of compensatory growth. Therefore, we focused on the early stage of refeeding and reduced the time point of metabolome analysis according to the results of transcription.

When the zebrafish were refed for 3 days (R1) and 10 days (R2) after 3 weeks of fasting, the liver tissues were quickly obtained and frozen in liquid nitrogen. The livers of continuously fed zebrafish were set as the control group. Ten liver tissues were mixed as one sample for the metabolomics analysis, and 10 samples were prepared for each time point.

The liver samples were quenched with methanol as a ratio of 40 mg: 600 μl, grinded with a high flux organization (70 Hz, 1 min). 36 μl heptadecanoic acid (0.2 mg/mL stock in methanol) was added as internal quantitative standard and vortex for 30 s. Then the samples were sonicated for 30 min at room temperature and stewed for 30 min on the ice. The sonicated samples were centrifuged at 14,000 g at 4 °C for 10 min and blow-dried by vacuum concentration. The residues were resuspended and derivatized for 2 h at 37 °C in 75 μl 15 mg/ml methoxyamine hydrochloride in pyridine, followed by 30 min treatment with 60 μl O-bis (trimethylsilyl) trifluoroacetamide (BSTFA) at 37 °C. The samples were then centrifuged at 12,000 g at 4 °C for 10 min, and the supernatant were subjected to GC-MS analysis.

The samples were injected into an Agilent 7890A/5975C GC-MS system (Agilent, US). 1 μl sample was injected into a 30 m × 250 μm i.d. × 0.25 μm DBS-MS capillary column using auto sampler. The initial temperature of the GC oven was held at 60 °C for 2 min followed by an increase to 300 °C at a rate of 10 °C /min then held for 5 min. Helium was used as carrier gas and flow was kept constant at 1 mL/min. The MS was determined by full-scan method with range from 35 to 750 m/z. Analysis was carried out by Agilent 7890A GC equipped with an Agilent 5975C VL MSD detector (Agilent Technologies).

The raw data were converted into netCDF format by Agilent MSD ChemStation workstation. Peak identification, peak filtration and peaks alignment were performed by using R (v3.1.3) XCMS program package. A file containing mass to charge ratio (m/z), retention time, and intensity was obtained. The metabolites were annotated with the AMDIS program, the database used for the annotation including the National Institute of Standards and Technology (NIST) Mass Spectral Library and Wiley Registry Metabolome Database, while the alkanes retention index was calculated based on the retention index provided by The Golm Metabolome Database (GMD) (http://gmd.mpimp-golm.mpg.de/) for further material characterization. Most of the substances were further confirmed by the standard and exported to excel for subsequent analysis. In order to compare the data among different magnitudes, internal standard normalization or total peak area normalization was performed on the data.

Multivariate statistical analysis was performed with SIMCA-P (Umetrics), PLS-DA was performed using centered scaling. *p*-value ≤0.05 + fold change ≥1.5 or ≤ 0.667, while one-way ANOVA *p*-value ≤0.05 were used to determine metabolites with changes in concentration. Z-score analysis scaled each metabolite according to a reference distribution and calculated based on the mean and standard deviation of reference sets control. Box plot was performed in the R platform with the package gplots (http://cran.r-project.org/src/contrib/Descriptions/gplots.html) using the distance matrix.

## Supplementary information

**Additional file 1.** Cluster I gene lists and GO analysis.

**Additional file 2.** Cluster II gene lists and GO analysis.

**Additional file 3.** Cluster III gene lists and GO analysis.

**Additional file 4.** Cluster IV gene lists and GO analysis.

**Additional file 5.** Tables of metabolite changes in zebrafish liver after 3 days and 10 days refeeding.

**Additional file 6.** Primer sequences used for quantitative real-time PCR (qPCR).

## Data Availability

Raw data of gene expression study is available at Gene Expression Omnibus (https://www.ncbi.nlm.nih.gov/geo/query/acc.cgi?acc=GSE112272) with the accession number of GSE112272.

## Abbreviations

2PG: Glycerol-2-phosphate
3PG: Glycerol-3-phosphate
AgrP: Agouti gene-related Protein
DAVID: Database for Annotation, Visualization and Integrated Discovery
GAPDH: Glyceraldehyde-3-phosphate dehydrogenase
GC-MS: Gas Chromatography-Mass Spectrometer
GO: Gene Ontology
GPCRs: G protein-coupled receptors
HRP: Horse Radish Peroxidase
IGF1: Insulin-like Growth Factor 1
NPY: Neural Peptide Y
ORs: Odorant receptors
PCA: Principal-Component Analysis
PCNA: Proliferating-cell nuclear antigen
PLS-DA: Partial least squares discriminant analysis
T3: triiodothyronine
TCA cycle: Tricarboxylic acid cycle
TG: Triglyceride
TH: Thyroid hormone
